# Plant‐based diets could ameliorate the risk factors of cardiovascular diseases in adults with chronic diseases

**DOI:** 10.1002/fsn3.3164

**Published:** 2022-12-08

**Authors:** Mostafa Lotfi, Mehran Nouri, Abduladheem Turki Jalil, Abbas Rezaianzadeh, Siavash Babajafari, Masoumeh Ghoddusi Johari, Shiva Faghih

**Affiliations:** ^1^ Department of Community Nutrition, School of Nutrition and Food Science Shiraz University of Medical Sciences Shiraz Iran; ^2^ Health Policy Research Center, Institute of Health Shiraz University of Medical Sciences Shiraz Iran; ^3^ Students' Research Committee Shiraz University of Medical Sciences Shiraz Iran; ^4^ Medical Laboratories Techniques Department Al‐Mustaqbal University College, Babylon Hilla Iraq; ^5^ Department of Epidemiology, School of Health and Nutrition Shiraz University of Medical Sciences Shiraz Iran; ^6^ Nutrition research center, Department of Clinical Nutrition School of Nutrition and Food Science, Shiraz University of Medical Sciences Shiraz Iran; ^7^ Breast Diseases Research Center Shiraz University of Medical Sciences Shiraz Iran; ^8^ Nutrition Research Center Shiraz University of Medical Sciences Shiraz Iran

**Keywords:** cardiovascular disease risk factors, dietary pattern, plant‐based diet

## Abstract

Adherence to plant‐based diets is recommended to prevent and control chronic diseases. However, not all plant‐based foods are healthy for this purpose. This study investigated the relationship between plant‐based diets and risk factors for cardiovascular diseases (CVDs) in adults with chronic diseases. This cross‐sectional study was performed on 3678 males and females (age range: 40–70 years) with chronic diseases who participated in the Kharameh cohort study. A validated semiquantitative food‐frequency questionnaire was used to calculate the plant‐based diet index (PDI), healthy plant‐based diet index (hPDI), and unhealthy plant‐based diet index (uPDI). Lipid profile, fasting blood sugar (FBS), blood pressure, and anthropometric indices were measured. Multivariable‐adjusted logistic regression analysis was performed to determine the association between plant‐based diets and CVDs risk factors. Higher adherence to the PDI was inversely associated with the level of FBS (odds ratio [OR] = 0.42; 95% confidence interval [CI]: 0.33–0.53; *p* < .001). A significant decrease was observed for total cholesterol in those with higher adherence to hPDI (OR = 0.80; 95% CI: 0.65–0.98; *p* = .035). Additionally, the score of uPDI was positively related to FBS (OR = 1.23; 95% CI: 1.00–1.53; *p* = .01), total cholesterol (OR = 1.23; 95% CI: 1.01–1.49; *p* = .061), and low‐density lipoprotein (OR = 1.39; 95% CI: 1.13–1.71; *p* = .009). It was concluded that adherence to PDI and hPDI was related to a lower level of FBS and total cholesterol, respectively. Moreover, the findings suggested that regular intake of the uPDI was correlated with some risk factors for CVDs in adults with chronic diseases.

## Graphical abstract

It was concluded that adherence to the PDI and hPDI was related to decreasing FBS and TC, respectively. Moreover, the findings suggested that regular intake of the uPDI was correlated with some risk factors of CVDs in adults with chronic diseases.

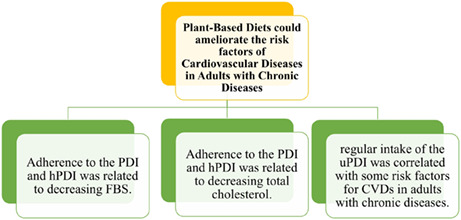

## 1 INTRODUCTION

Cardiovascular diseases (CVDs) are among the leading causes of mortality and the main causes of health system costs (Abdulmuhsin, 2016). In addition, the prevalence of CVDs has increased in recent decades. Numerous investigations have shown that the control of CVD risk factors, such as blood pressure, blood lipids, obesity, and blood sugar, could lead to the improvement of patients’ complications (Khunti et al., 2018; Randhawa et al., 2020; Shmakova et al., 2022).

Lifestyle risk factors, such as smoking, inadequate physical activity, alcohol consumption, and unhealthy diets, are modifiable and known factors for the control of some chronic noncommunicable diseases (Bauman, 2004; Farazian et al., 2019; Wu et al., 2015). Diet plays a key role in the incidence and control of chronic diseases. Various studies on the relationship of plant‐ and animal‐based diets with chronic diseases have reported different results. Some studies have shown that restricting animal foods and consuming more plant‐based foods could reduce body mass index (BMI), blood pressure, blood sugar, or inflammation (Hematdar et al., 2018; Picasso et al., 2019; Yokoyama et al., 2014). However, another study showed that the moderate consumption of animal foods, such as red meat, has not adversely affected cardio‐metabolic factors (Hassanzadeh‐Rostami et al., 2021), and some others have not reported any association (Chiang et al., 2013; Vinagre et al., 2013; Yang et al., 2011).

In comparison to omnivorous diets, vegetarian food regimens are full of fibers, phytosterols, magnesium, Fe^3+^, folic acid, vitamins C and E, omega‐6 polyunsaturated fatty acids (PUFAs), phytochemicals, and antioxidants but low in total fat and saturated fatty acids (SFAs), sodium, Fe^2+^, zinc, vitamins A, B_12_, and D, and little or no cholesterol (Borazjani et al., 2022; Kamalipour & Akhondzadeh, 2011). Therefore, plant‐based diets could have numerous healthy effects (Jalilpiran et al., 2017; Wang et al., 2015). Recently, it has been known that not every plant food retains the same beneficial features. Some plant foods, such as refined grains and sweetened beverages, have adverse effects on health (Hemler & Hu, 2019). In general, due to the contradictory results and limitations of previous studies, the present study examined three types of plant‐based diet indices, including the general plant‐based diet index (PDI), healthy plant‐based diet index (hPDI), and unhealthy plant‐based diet index (uPDI), and their relationships with blood lipids, blood pressure, glycemic control, and anthropometric indices in patients with chronic disease.

## 2 METHODS

### 2.1 Study design and study population

The present cross‐sectional study was carried out on the information of participants of the Kharameh cohort study. The Kharameh cohort study is a branch of Prospective Epidemiological Research Studies in Iran (PERSIAN) (Poustchi et al., 2018) in which 10,663 individuals aged 40–70 years were enrolled from 2014 to 2017 (Rezaianzadeh et al., 2021). Demographic information, physical activity, smoking status, and medical history of the participants were assessed in the PERSIAN cohort study. Additionally, physical examinations (i.e., weight, height, waist circumference [WC], hip circumference [HC], systolic blood pressure [SBP], and diastolic blood pressure [DBP]), biochemical assessments (i.e., fasting blood sugar [FBS], total cholesterol, triglyceride [TG], high‐density lipoprotein [HDL], low‐density lipoprotein [LDL], and alkaline phosphatase), and dietary evaluation were performed (Nikbakht et al., 2020).

Inclusion criteria for the Kharameh cohort study were the age of 40–70 years, living in Kharameh for the last 9 months, and having Iranian nationality. In addition, having a history of one or more types of CVDs (heart failure, angina, and myocardial infarction), hypertension, or diabetes was needed to be included in the present study.

**FIGURE 1:**
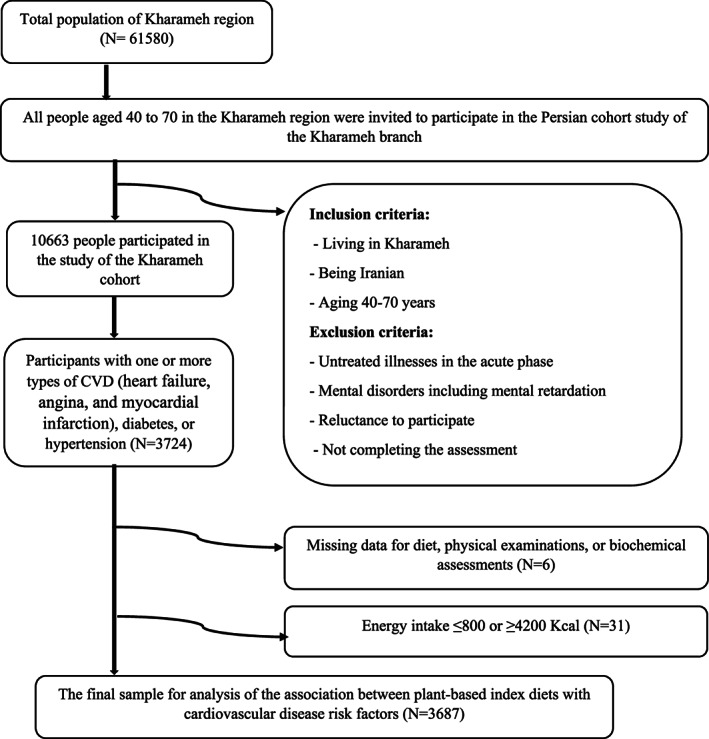
Flow diagram of the study.

Participants with mental disorders or untreated illnesses in the acute phase and also who did not complete the assessments were excluded from the study. Furthermore, we excluded the data of the participants who under‐ or over‐reported their energy intakes (<800 kcal or > 4200 kcal/day) (Figure 1). This study was approved by the Ethics Committee of Shiraz University of Medical Sciences, Fars, Iran (code: IR.SUMS.REC.1399.1115).

### 2.2 Dietary intake and plant‐based diet assessment

Dietary intakes were collected using a 130‐item semiquantitative food‐frequency questionnaire (FFQ). Consistent with household measures, the records from completed FFQs were transformed into grams. Then, energy and nutrient intakes were calculated using the adapted version of Nutritionist IV for Iranian software (version 7.0; N‐Squared Computing).

In this study, Satije et al. approach was used to calculate the PDI, hPDI, and uPDI (Satija et al., 2016). A total of 130 food items were categorized into 18 food groups; then, the food groups were divided into three principal categories, including healthy plant foods (i.e., whole grains, nuts, vegetables, vegetable oils, fruits, legumes, and tea/coffee), unhealthy plant foods (i.e., refined grains, sugar‐sweetened drinks, fruit juices, potatoes, and sweets/desserts), and animal foods (i.e., egg, dairy, fish/kinds of seafood, animal fats, meat, and miscellaneous animal‐based foods). In the PDI and hPDI, the maximum and minimum intakes of plant foods and healthy plant foods received scores of 10 and 1, respectively. In the uPDI, scores of 1 and 10 were considered for maximum and minimum intakes of unhealthy plant foods, respectively. Rankings were summed up to get a score ranging from 18 to 180 for each of the PDI, hPDI, and uPDI. Overall, a higher score for each index indicated higher adherence to that dietary pattern.

### 2.3 Anthropometric and biochemical assessments

The trained workforce measured participants’ height, weight, WC, HC, and blood pressure. Height, weight, HC, and WC were measured to the nearest 0.1 cm or 0.1 kg in light clothing with no shoes. BMI was calculated by dividing the weight by height squared. Blood pressure was measured after a 10‐min rest in the sitting position using a standard calibrated sphygmomanometer (Riester Model, Germany). For laboratory assessments, after 10–14 h overnight fast, a 20‐ml blood sample was taken from each participant. Serum separation was done in a minimum of 30 min or a maximum of 2 h. Then, 0.5 ml of serum was used to do the biochemical tests, and the remaining was transferred to cryotubes for storage at −80°C until further analyses. Serum FBS, TG, and cholesterol were measured using the Mindray BS‐380 device by Pars Azmoon kits. HDL, TG, and total cholesterol levels were determined using an enzymatic technique, and LDL level was calculated by Friedewald formula (Friedewald et al., 1972).

### 2.4 Covariates

We considered age, gender, physical activity, educational level, and smoking status as covariates. Demographic characteristics, including age, gender, educational level, and smoking status of the participants, were obtained by a questionnaire. The duration of the participants’ education was asked to determine their educational level. The participants’ smoking status was determined by answering yes or no. Participants’ daily physical activity was assessed by a self‐reported validated questionnaire. Participants reported the time spent on all activities such as sleeping, running, and walking on a typical day (24 h), during the previous year. Each activity was given a value in metabolic equivalent tasks (METs), and then total metabolic equivalent tasks per day were computed (Kazemi Karyani et al., 2019).

### 2.5 Statistical analyses

BMI ≥30 kg/m^2^, SBP ≥135 mmHg, DBP ≥85 mmHg, FBS ≥126 mg/dl, TG ≥150 mg/dl, total cholesterol ≥200 mg/dl, LDL‐C ≥ 130 mg/dl, HDL‐C ˂ 40 mg/dl for men and 50 mg/dl for women, TG to HDL ratio ≥5, and total cholesterol to HDL ratio ≥3 were considered as abnormal levels (Huang, 2009; Kohansal et al., 2022; Salcedo‐Cifuentes et al., 2020). All data were analyzed using SPSS for Windows software (version 20.0), and a *p*‐value less than .05 was considered statistically significant. The Kolmogorov–Smirnov test was used to examine the normal distribution of the variables. For comparing the baseline characteristics between the male and female patients, an independent‐samples *t*‐test and chi‐squared test were used for continuous variables and categorical variables, respectively. One‐way analysis of variance was used to compare the intake of nutrients and food groups across the quartiles of plant‐based diet indices (i.e., PDI, hPDI, and uPDI). Two different multivariable logistic regression models were used to assess the relationship between plant‐based diet indices and odds of CVD risk factors. Gender, age, physical activity, total energy intake, and smoking status were included in the regression models as confounders.

## 3 RESULTS

The data of 3687 participants with one or more types of CVDs, hypertension, or diabetes were included in the analysis (Figure 1). Table 1 shows the baseline characteristics of the participants. Male participants had higher age (*p* = .01), educational level (*p* < .001), weight (*p* < .001), height (*p* < .001), and physical activity (*p* = .004). However, female participants had higher WC, HC, and waist‐to‐hip ratios (*p* < .001 for all).

**TABLE 1:** Baseline characteristics of the participants.

Variables	Male	Female	*p* value
Age (years)	56.20 ± 7.74	55.50 ± 8.01	**.01**
Education (years)	5.56 ± 5.01	2.48 ± 3.24	**<.001**
Weight (kg)	74.55 ± 12.46	68.02 ± 11.64	**<.001**
Height (cm)	170.36 ± 6.77	156.46 ± 5.55	**<.001**
Waist circumference (cm)	97.50 ± 11.43	99.67 ± 11.34	**<.001**
Hip circumference (cm)	100.89 ± 7.29	102.19 ± 8.97	**<.001**
WHR	0.96 ± 0.06	0.97 ± 0.07	**<.001**
Physical activity (Met. h/day)	37.74 ± 7.64	36.87 ± 3.31	**.004**
Smoking			
Yes	571 (84.7%)	103 (15.3%)	**<.001**
No	603 (20%)	2410 (80%)	

Abbreviations: FBS, fasting blood glucose; HDL, high‐density lipoprotein; LDL, low‐density lipoprotein; TC, total cholesterol; TG, triglyceride; WHR, waist to hip ratio.

*Note*: Values are mean ± standard deviation (SD) or number (percent). *p*‐value less than .05 was considered significant. Independent‐samples t‐test for continuous and chi‐squared test for categorical variables was done.

All *p* values and *p* trends are bolded.

According to Table 2, the participants in the highest PDI quartiles had higher intakes of carbohydrates, fiber, omega‐6/omega‐3 fatty acid (FA), beta carotene, vitamin E, C, B_9_, potassium, and magnesium (*p* < .001 for all, except for vitamins E and B_9_). In addition, the participants in the highest PDI quartiles had lower intakes of energy, protein, fat, cholesterol, SFAs, monounsaturated fatty acids (MUFAs), calcium, and selenium (*p* < .001 for all).

**TABLE 2:** Dietary intakes of participants across the quartiles of PDI, hPDI, uPDI scores.

Food groups	PDI				hPDI					uPDI					
	Q1	Q2	Q3	Q4	*p* value	Q1	Q2	Q3	Q4	*p* value	Q1	Q2	Q3	Q4	*p* value
Energy (Kcal/d)	2432.29 ± 20.11	2116.28 ± 21.38	2081.52 ± 21.73	2165.77 ± 21.65	**<.001**	2147.11 ± 19.95	2059.54 ± 21.05	2223.67 ± 20.89	2480.36 ± 10.88	**<.001**	2233.15 ± 20.68	2097.04 ± 22.02	2109.58 ± 20.68	2393.72 ± 22.31	**<.001**
CHO (gr/d)	354.06 ± 0.94	364.48 ± 0.91	370.77 ± 0.80	379.58 ± 0.94	**<.001**	363.26 ± 0.81	365.90 ± 0.86	367.36 ± 0.99	371.78 ± 1.24	**<.001**	359.68 ± 0.98	363.75 ± 0.80	367.75 ± 0.90	376.27 ± 1.02	**<.001**
Protein (gr/d)	76.21 ± 0.32	72.15 ± 0.30	69.16 ± 0.26	65.14 ± 0.31	**<.001**	72.51 ± 0.27	70.63 ± 0.29	70.57 ± 0.34	69.26 ± 0.43	**<.001**	72.96 ± 0.32	71.96 ± 0.30	69.78 ± 0.32	68.68 ± 0.35	**<.001**
Fat (gr/d)	58.02 ± 0.38	55.66 ± 0.38	54.45 ± 0.33	52.75 ± 0.38	**<.001**	55.42 ± 0.32	55.39 ± 0.35	55.45 ± 0.39	55.07 ± 0.48	0.90	58.30 ± 0.39	56.43 ± 0.31	55.33 ± 0.36	50.90 ± 0.39	**<.001**
Fiber (gr/d)	24.32 ± 0.19	25.01 ± 0.21	25.33 ± 0.18	25.76 ± 0.20	**<.001**	23.31 ± 0.18	23.75 ± 0.16	25.84 ± 0.17	28.55 ± 0.25	**<.001**	26.82 ± 0.20	25.64 ± 0.17	24.77 ± 0.19	22.78 ± 0.20	**<.001**
Cholesterol (mg/d)	251.88 ± 3.01	209.54 ± 2.68	187.23 ± 2.20	159.00 ± 2.27	**<.001**	232.78 ± 2.68	211.78 ± 2.61	192.35 ± 2.51	164.84 ± 3.15	**<.001**	222.70 ± 2.99	217.79 ± 2.51	201.92 ± 2.65	171.75 ± 2.83	**<.001**
Trans fat (mg/d)	0.23 ± 0.007	0.21 ± 0.006	0.21 ± 0.005	0.21 ± 0.007	.22	0.25 ± 0.006	0.22 ± 0.006	0.20 ± 0.006	0.18 ± 0.007	**<.001**	0.23 ± 0.006	0.23 ± 0.007	0.22 ± 0.007	0.18 ± 0.006	**<.001**
SFA (gr/d)	20.65 ± 0.19	19.54 ± 0.19	18.81 ± 0.17	18.03 ± 0.19	**<.001**	19.44 ± 0.16	19.50 ± 0.18	19.43 ± 0.19	18.78 ± 0.24	.05	20.43 ± 0.19	19.57 ± 0.17	19.43 ± 0.19	17.69 ± 0.20	**<.001**
MUFA (gr/d)	16.40 ± 0.16	15.64 ± 0.16	15.37 ± 0.14	15.00 ± 0.16	**<.001**	15.66 ± 0.13	15.67 ± 0.14	15.63 ± 0.16	15.59 ± 0.20	.98	16.96 ± 0.16	16.21 ± 0.13	15.63 ± 0.14	13.57 ± 0.16	**<.001**
PUFA (gr/d)	9.14 ± 0.11	9.10 ± 0.11	9.05 ± 0.10	8.99 ± 0.11	.77	8.94 ± 0.09	8.83 ± 0.10	9.02 ± 0.11	9.65 ± 0.14	**<.001**	10.06 ± 0.11	9.53 ± 0.09	9.00 ± 0.10	7.54 ± 0.11	**<.001**
Omega 6/3	15.52 ± 0.51	18.66 ± 0.59	24.49 ± 0.96	33.28 ± 1.57	**<.001**	18.22 ± 0.81	21.31 ± 0.95	23.19 ± 0.74	30.58 ± 1.50	**<.001**	21.64 ± 1.13	20.50 ± 0.65	24.07 ± 1.08	24.51 ± 0.94	**.01**
Beta carotene (IU/d)	4527.90 ± 80.33	4758.50 ± 85.89	4810.83 ± 81.01	5103.98 ± 109.77	**<.001**	4293.87 ± 66.06	4433.73 ± 74.17	4932.61 ± 79.71	5823.78 ± 141.35	**<.001**	5380.57 ± 87.84	4967.06 ± 77.13	4783.80 ± 103.53	3919.23 ± 78.18	**<.001**
Vit E (mg/d)	7.11 ± 0.07	7.18 ± 0.07	7.28 ± 0.07	7.43 ± 0.08	**.01**	6.93 ± 0.05	7.02 ± 0.06	7.31 ± 0.07	7.93 ± 0.10	**<.001**	7.77 ± 0.07	7.58 ± 0.06	7.24 ± 0.07	6.29 ± 0.07	**<.001**
Vit c (mg/d)	109.95 ± 1.57	119.07 ± 1.93	120.03 ± 1.70	126.74 ± 1.87	**<.001**	108.83 ± 1.40	112.97 ± 1.61	121.61 ± 1.70	136.69 ± 2.45	**<.001**	131.48 ± 1.76	126.05 ± 1.66	117.68 ± 1.67	96.63 ± 1.72	**<.001**
Vit B9 (mcg/d)	561.47 ± 3.25	561.12 ± 3.09	569.26 ± 2.96	571.67 ± 3.49	**.04**	551.30 ± 2.87	554.02 ± 2.75	571.66 ± 3.01	596.14 ± 4.29	**<.001**	562.39 ± 3.01	558.40 ± 2.91	563.79 ± 3.35	579.05 ± 3.54	**<.001**
K (mcg/d)	3260.77 ± 25.15	3332.48 ± 28.20	3340.08 ± 24.48	3440.74 ± 28.13	**<.001**	3170.34 ± 21.95	3212.42 ± 22.69	3405.30 ± 25.61	3681.40 ± 35.27	**<.001**	3597.82 ± 26.82	3447.61 ± 21.24	3319.26 ± 25.60	2950.46 ± 26.31	**<.001**
Ca (mg/d)	1090.86 ± 6.83	1056.69 ± 6.97	1022.08 ± 6.23	958.77 ± 6.47	**<.001**	1034.62 ± 5.91	1026.73 ± 6.35	1044.79 ± 6.92	1033.70 ± 8.84	.31	1032.07 ± 6.51	1022.03 ± 6.32	1025.48 ± 6.75	1061.00 ± 7.72	**<.001**
Mg (mg/d)	318.36 ± 1.56	322.50 ± 1.70	323.79 ± 1.51	328.31 ± 1.85	**<.001**	312.26 ± 1.40	313.70 ± 1.30	326.43 ± 1.52	347.40 ± 2.31	**<.001**	338.38 ± 1.66	327.96 ± 1.29	321.46 ± 1.75	301.79 ± 1.59	**<.001**
Se (mcg/d)	107.05 ± 0.69	100.02 ± 0.65	97.27 ± 0.61	90.75 ± 0.67	**<.001**	102.38 ± 0.58	99.72 ± 0.61	97.48 ± 0.71	95.69 ± 0.91	**<.001**	97.95 ± 0.63	98.26 ± 0.62	97.82 ± 0.66	102.92 ± 0.83	**<.001**

Abbreviations: Ca, calcium; hPDI, healthy plant‐based diet index; K, potassium; Mg, magnesium; MUFA, monounsaturated fatty acid; PDI, plant‐based diet index; PUFA, polyunsaturated fatty acid; Se, selenium; SFA, saturated fatty acid; uPDI, unhealthy plant‐based diet index; Vit, vitamin.

*Note*: Values are mean ± standard error. *p* value less than .05 was considered significant. One‐way ANOVA has been used.

All *p* values and *p* trends are bolded.

The highest consumption of energy, carbohydrate, fiber, omega‐6/omega‐3 FA, PUFAs, beta carotene, vitamin E, vitamin C, vitamin B_9_, potassium, and magnesium and the lowest consumption of protein, cholesterol, trans fatty acids, and selenium were observed in the highest hPDI quartile (*p* < .001 for all). Moreover, the highest intake of energy, carbohydrate, omega‐6/omega‐3 FA, vitamin B_9_, calcium, and selenium and the lowest intake of protein, fat, fiber, cholesterol, trans fatty acids, SFAs, MUFAs, PUFAs, beta carotene, vitamin E, vitamin C, potassium, and magnesium were observed in the highest uPDI quartile (*p* < .001 for all, except for omega‐6/omega‐3 FA) (Table 2).

Adherence to the PDI was significantly associated with higher consumption of fruits, vegetables, legumes, vegetable oils, tea/coffee, fruit juices, potatoes, sugar‐sweetened beverages, and sweet desserts and lower consumption of animal fat, dairy, egg, fish/seafood, meat, and animal‐based foods (*p* < .001 for all, except for fruits and fruit juices). Furthermore, adherence to the hPDI was significantly associated with higher consumption of whole grains, fruits, vegetables, nuts, legumes, vegetable oils, and tea/coffee and lower consumption of refined grains, potatoes, sugar‐sweetened beverages, sweet desserts, animal fat, dairy, egg, fish/seafood, meat, and animal‐based foods (*p* < .001 except for dairy and fish/seafood). In addition, the participants in the highest quartile of the uPDI had higher levels of refined grains (*p* < .001), potatoes (*p* = .03), sugar‐sweetened beverages (*p* = .009), and sweet desserts (*p* < .001) and lower intakes of whole grains, fruits, vegetables, nuts, legumes, vegetable oils, animal fat, dairy, egg, fish/seafood, meat, and animal‐based foods (*p* < .001 for all, except for vegetable oils) (Table 3).

**TABLE 3:** Mean and standard error of food groups intakes across the quartiles of PDI, hPDI, uPDI scores.

Food groups	PDI				hPDI					uPDI					
	Q1	Q2	Q3	Q4	*p* value	Q1	Q2	Q3	Q4	*p* value	Q1	Q2	Q3	Q4	*p* value
Whole grains (g/d)	32.03 ± 2.20	32.71 ± 2.01	34.63 ± 1.86	33.55 ± 1.66	.79	27.73 ± 1.62	25.33 ± 1.20	30.59 ± 1.79	55.35 ± 3.30	**<.001**	45.12 ± 2.14	35.10 ± 1.81	31.14 ± 1.99	19.68 ± 1.79	**<.001**
Fruits (g/d)	304.18 ± 5.81	321.31 ± 6.62	326.98 ± 5.84	332.66 ± 6.23	**.005**	284.73 ± 5	296.47 ± 5.24	339.16 ± 6.14	383.83 ± 8.25	**<.001**	362.68 ± 6.23	344.22 ± 5.69	317.36 ± 5.92	251.15 ± 5.83	**<.001**
Vegetables (g/d)	439.38 ± 6.72	469.11 ± 8.47	469.75 ± 6.40	510.58 ± 7.70	**<.001**	419.53 ± 6.47	440.66 ± 6.40	493.72 ± 6.77	558.25 ± 9.33	**<.001**	515.44 ± 6.99	488.97 ± 6.60	468.87 ± 7.27	400.80 ± 7.78	**<.001**
Nuts (g/d)	3.70 ± 0.18	3.83 ± 0.15	3.99 ± 0.15	4.14 ± 0.18	.28	3.13 ± 0.11	3.65 ± 0.16	4.13 ± 0.18	5.17 ± 0.25	**<.001**	4.77 ± 0.17	4.37 ± 0.17	3.86 ± 0.16	2.48 ± 0.16	**<.001**
Legumes (g/d)	26.11 ± 0.71	27.72 ± 0.70	29.70 ± 0.63	30.90 ± 0.72	**<.001**	26.61 ± 0.58	26.34 ± 0.57	29.61 ± 0.68	32.84 ± 1.01	**<.001**	32.51 ± 0.74	29.24 ± 0.63	27.37 ± 0.66	24.29 ± 0.69	**<.001**
Vegetable oils (g/d)	14.38 ± 0.30	15.32 ± 0.31	16.43 ± 0.30	17.95 ± 0.33	**<.001**	14.42 ± 0.25	16.13 ± 0.30	16.46 ± 0.32	17.44 ± 0.41	**<.001**	16.13 ± 0.30	15.94 ± 0.31	16.46 ± 0.33	15.21 ± 0.31	**.04**
Tea and coffee (g/d)	583.25 ± 16.27	673.72 ± 18.80	754.14 ± 16.95	929.77 ± 22.01	**<.001**	664.60 ± 13.97	728.93 ± 17.90	738.85 ± 18.88	810.25 ± 27.03	**<.001**	740.66 ± 20.40	707.97 ± 17.44	738.25 ± 17.93	721.21 ± 18.78	.57
Fruit juices (g/d)	1.35 ± 0.25	2.54 ± 0.35	2.32 ± 0.28	2.17 ± 0.28	**.01**	2.42 ± 0.26	2.28 ± 0.28	1.55 ± 0.22	1.85 ± 0.41	.12	2.24 ± 0.29	2.55 ± 0.31	1.87 ± 0.26	1.55 ± 0.28	.08
Refined grains (g/d)	485.74 ± 4.68	484.65 ± 4.90	481.18 ± 4.38	473.98 ± 4.66	.29	493.57 ± 3.91	495.18 ± 4.27	481.72 ± 4.59	445.13 ± 6.24	**<.001**	433.62 ± 4.57	466.47 ± 3.60	485.38 ± 4.26	548.25 ± 2.19	**<.001**
Potatoes (g/d)	20.29 ± 0.72	23.27 ± 0.77	26.59 ± 0.77	33.27 ± 0.84	**<.001**	29.76 ± 0.76	27.77 ± 0.74	23.73 ± 0.74	18.50 ± 0.85	**<.001**	24.01 ± 0.74	25.51 ± 0.72	25.75 ± 0.81	27.26 ± 0.87	**.03**
Sugar‐sweetened beverages (g/d)	52.45 ± 2.56	60.51 ± 2.97	60.79 ± 2.33	80.09 ± 3.31	**<.001**	83.38 ± 2.72	71.10 ± 2.88	49.49 ± 2.22	36.47 ± 3	**<.001**	55.14 ± 2.22	64.88 ± 3.03	66.78 ± 2.78	65.32 ± 3.16	**.009**
Sweet desserts (g/d)	29.19 ± 1.12	37.09 ± 1.26	44.14 ± 1.22	54.61 ± 1.33	**<.001**	43.01 ± 1.02	44.88 ± 1.18	38.94 ± 1.24	33.85 ± 1.74	**<.001**	36.58 ± 1.18	38.58 ± 1.02	42.90 ± 1.19	45.32 ± 1.59	**<.001**
Animal fat (g/d)	2.40 ± 0.13	1.64 ± 0.10	1.27 ± 0.07	0.77 ± 0.05	**<.001**	1.89 ± 0.09	1.87 ± 0.10	1.53 ± 0.10	0.68 ± 0.08	**<.001**	1.96 ± 0.11	1.68 ± 0.09	1.58 ± 0.09	0.97 ± 0.09	**<.001**
Dairy (g/d)	264.01 ± 4.76	241.75 ± 4.90	206.85 ± 4.12	169.55 ± 3.72	**<.001**	231.86 ± 3.93	220.75 ± 4.32	224.99 ± 4.76	206.57 ± 5.72	**.002**	250.48 ± 4.70	233.58 ± 4.33	219.74 ± 4.74	181.79 ± 4.21	**<.001**
Egg (g/d)	26.79 ± 0.62	20.17 ± 0.59	17.16 ± 0.47	12.63 ± 0.43	**<.001**	24.83 ± 0.53	20.85 ± 0.56	16.84 ± 0.49	13.03 ± 0.62	**<.001**	21.51 ± 0.60	21.77 ± 0.56	18.95 ± 0.53	15.66 ± 0.54	**<.001**
Fish or sea foods (g/d)	4.16 ± 0.20	3.32 ± 0.19	2.67 ± 0.15	1.83 ± 0.13	**<.001**	3.55 ± 0.15	3.04 ± 0.16	2.86 ± 0.17	2.52 ± 0.22	**.001**	4.22 ± 0.19	3.28 ± 0.17	2.70 ± 0.17	1.81 ± 0.15	**<.001**
Meats (g/d)	64 ± 1.07	54.20 ± 1.03	47.60 ± 0.84	41.21 ± 0.92	**<.001**	57.24 ± 0.90	53.76 ± 0.95	50.82 ± 1.06	44.54 ± 1.18	**<.001**	57.72 ± 1.01	53.13 ± 0.94	50.29 ± 0.99	44.23 ± 1.06	**<.001**
Animal‐based foods (g/d)	4.16 ± 0.19	3.29 ± 0.17	2.77 ± 0.11	2.31 ± 0.14	**<.001**	3.90 ± 0.16	3.65 ± 0.18	2.82 ± 0.14	1.89 ± 0.14	**<.001**	3.79 ± 0.19	3.51 ± 0.16	3.27 ± 0.16	2.04 ± 0.12	**<.001**

Abbreviations: hPDI, healthy plant‐based diet index; PDI, plant‐based diet index; uPDI, unhealthy plant‐based diet index. Values are mean ± standard error. *p* value less than .05 was considered significant; one‐way ANOVA has been used.

All *p* values and *p* trends are bolded.

As shown in Table 4, adherence to the PDI was related to a lower level of FBS (*p* < .001) in the crude and adjusted models. However, there was no significant association between adherence to the PDI and any CVD risk factors. Additionally, after adjusting for gender, age, energy intake, smoking, and physical activity, higher scores of the hPDI were associated with a lower total cholesterol level (odds ratio [OR] = 0.80; 95% confidence interval [CI]: 0.65–0.98; *p* = .035) and cholesterol to HDL ratio (OR = 0.74; 95% CI: 0.57–0.95; *p* = .05). In the adjusted model, the scores of the uPDI were positively related to FBS (OR = 1.25; 95% CI: 1.01–1.55; *p* = .01), total cholesterol (OR = 1.24; 95% CI: 1.0–1.50; *p* = .05), and LDL (OR = 1.40; 95% CI: 1.13–1.72; *p* = .009).

**TABLE 4:** Crude and adjusted odds ratios and 95% CIs for lipid profile, blood pressure, fasting blood sugar, and BMI across quartiles of PDI, hPDI, and uPDI scores

Variables	PDI					hPDI					uPDI				
	Q1	Q2	Q3	Q4	*p* trend	Q1	Q2	Q3	Q4	*p* trend	Q1	Q2	Q3	Q4	*p* trend
BMI (k/m^2^)															
Crude	1.00	1.01 (0.83, 1.23)	1.07 (0.88, 1.29)	0.93 (0.76, 1.13)	.69	1.00	0.95 (0.79, 1.14)	1.12 (0.93, 1.35)	1.18 (0.96, 1.44)	.05	1.00	1.09 (0.90, 1.33)	0.92 (0.75, 1.11)	0.88 (0.73, 1.07)	.10
Adjusted	1.00	0.95 (0.77, 1.16)	0.96 (0.79, 1.18)	0.84 (0.69, 1.03)	.14	1.00	0.93 (0.77, 1.13)	1.14 (0.94, 1.38)	1.18 (0.95, 1.45)	**.04**	1.00	1.01 (0.83, 1.24)	0.87 (0.71, 1.06)	0.90 (0.74, 1.10)	.16
SBP (mmHg)															
Crude	1.00	0.96 (0.77, 1.21)	0.95 (0.77, 1.18)	1.08 (0.87, 1.34)	.57	1.00	1.20 (0.98, 1.48)	0.90 (0.72, 1.12)	0.89 (0.71, 1.13)	.13	1.00	1.13 (0.91, 1.40)	0.94 (0.75, 1.17)	1.01 (0.81, 1.26)	.74
Adjusted	1.00	0.94 (0.75, 1.19)	0.96 (0.77, 1.20)	1.08 (0.87, 1.35)	.21	1.00	1.16 (0.94, 1.43)	0.85 (0.69, 1.06)	0.87 (0.69, 1.10)	.07	1.00	1.16 (0.93, 1.44)	0.93 (0.74, 1.16)	1.00 (0.80, 1.25)	.62
DBP (mmHg)															
Crude	1.00	0.90 (0.68, 1.20)	0.92 (0.70, 1.20)	1.15 (0.88, 1.50)	.36	1.00	1.28 (0.99, 1.65)	0.97 (0.74, 1.27)	0.87 (0.65, 1.17)	.23	1.00	1.15 (0.88, 1.50)	0.90 (0.68, 1.19)	1.03 (0.78, 1.35)	.75
Adjusted	1.00	0.93 (0.70, 1.24)	0.96 (0.73, 1.26)	1.20 (0.92, 1.57)	.20	1.00	1.29 (1.00, 1.67)	0.97 (0.74, 1.27)	0.87 (0.65, 1.17)	.27	1.00	1.18 (0.90, 1.55)	0.92 (0.69, 1.21)	1.03 (0.78, 1.35)	.76
FBS(mg/dl)															
Crude	1.00	0.73 (0.60, 0.91)	0.56 (0.45, 0.69)	0.43 (0.34, 0.55)	**<.001**	1.00	0.71 (0.57, 0.87)	1.15 (0.94, 1.42)	1.06 (0.85, 1.33)	.09	1.00	0.90 (0.72, 1.13)	1.11 (0.89, 1.38)	1.23 (1.00, 1.53)	**.01**
Adjusted	1.00	0.71 (0.57, 0.87)	0.53 (0.43, 0.66)	0.42 (0.33, 0.53)	**<.001**	1.00	0.69 (0.55, 0.86)	1.13 (0.92, 1.38)	1.05 (0.84, 1.31)	.12	1.00	0.88 (0.70, 1.10)	1.09 (0.87, 1.35)	1.25 (1.01, 1.55)	**.01**
Triglycerides (mg/dl)															
Crude	1.00	1.01 (0.83, 1.22)	0.95 (0.79, 1.14)	1.01 (0.83, 1.22)	.91	1.00	0.90 (0.75, 1.09)	1.08 (0.90, 1.30)	1.15 (0.94, 1.40)	.07	1.00	0.99 (0.82, 1.20)	1.06 (0.88, 1.29)	0.90 (0.74, 1.09)	.49
Adjusted	1.00	1.01 (0.83, 1.23)	0.94 (0.78, 1.13)	1.01 (0.83, 1.22)	.87	1.00	0.91 (0.75, 1.09)	1.09 (0.90, 1.31)	1.16 (0.95, 1.41)	.06	1.00	0.97 (0.80, 1.17)	1.06 (0.88, 1.29)	0.90 (0.74, 1.10)	.55
Total cholesterol (mg/dl)															
Crude	1.00	1.16 (0.95, 1.40)	0.86 (0.71, 1.04)	1.06 (0.88, 1.29)	.74	1.00	0.87 (0.72, 1.05)	0.88 (0.73, 1.06)	0.80 (0.65, 0.98)	**.03**	1.00	1.17 (0.96, 1.41)	1.11 (0.91, 1.34)	1.23 (1.01, 1.49)	.06
Adjusted	1.00	1.07 (0.88, 1.31)	0.78 (0.65, 0.95)	0.96 (0.79, 1.17)	.18	1.00	0.84 (0.70, 1.01)	0.86 (0.72, 1.04)	0.79 (0.64, 0.96)	**.02**	1.00	1.13 (0.93, 1.38)	1.06 (0.88, 1.29)	1.24 (1.02, 1.50)	.05
LDLc (mg/dl)															
Crude	1.00	1.14 (0.92, 1.40)	0.91 (0.74, 1.20)	1.05 (0.85, 1.29)	.84	1.00	0.96 (0.79, 1.17)	0.89 (0.73, 1.09)	0.89 (0.72, 1.11)	.22	1.00	1.31 (1.06, 161)	1.15 (0.93, 1.42)	1.39 (1.13, 1.71)	**.009**
Adjusted	1.00	1.07 (0.87, 1.32)	0.85 (0.69, 1.05)	0.97 (0.79, 1.20)	.37	1.00	0.93 (0.76, 1.14)	0.88 (0.72, 1.07)	0.88 (0.71, 1.10)	.19	1.00	1.28 (1.04, 1.59)	1.11 (0.90, 1.38)	1.40 (1.13, 1.72)	**.009**
HDLc (mg/dl)															
Crude	1.00	0.84 (0.68, 1.04)	0.87 (0.72, 1.06)	0.90 (0.73, 1.10)	.32	1.00	0.95 (0.78, 1.16)	1.06 (0.87, 1.29)	1.09 (0.88, 1.34)	.29	1.00	0.86 (0.70, 1.06)	0.89 (0.73, 1.10)	0.89 (0.73, 1.09)	.32
Adjusted	1.00	1.01 (0.81, 1.25)	1.07 (0.87, 1.32)	1.13 (0.91, 1.40)	.22	1.00	1.05 (0.85, 1.29)	1.14 (0.93, 1.40)	1.15 (0.92, 1.43)	.13	1.00	0.90 (0.73, 1.12)	0.99 (0.80, 1.22)	0.87 (0.70, 1.08)	.34
TG/HDL ratio															
Crude	1.00	0.91 (0.70, 1.17)	0.71 (0.71, 1.16)	0.96 (0.74, 1.24)	.72	1.00	0.78 (0.60, 1.01)	1.01 (0.79, 1.29)	1.14 (0.88, 1.47)	.18	1.00	0.99 (0.77, 1.28)	1.07 (0.83, 1.38)	0.94 (0.73, 1.22)	.85
Adjusted	1.00	1.01 (0.78, 1.31)	1.01 (0.78, 1.30)	1.08 (0.83, 1.40)	.57	1.00	0.82 (0.64, 1.07)	1.06 (0.83, 1.35)	1.18 (0.91, 1.54)	.13	1.00	1.00 (0.77, 1.29)	1.13 (0.88, 1.46)	0.93 (0.72, 1.21)	.90
Cholesterol/HDL ratio															
Crude	1.00	0.98 (0.77, 1.25)	0.89 (0.71, 1.12)	0.80 (0.63, 1.01)	.05	1.00	0.75 (0.60, 0.94)	0.89 (0.71, 1.13)	0.78 (0.61, 1.00)	.14	1.00	1.14 (0.91, 1.44)	1.06 (0.84)	1.29 (1.02, 1.63)	.06
Adjusted	1.00	1.11 (0.87, 1.43)	1.01 (0.80, 1.28)	0.90 (0.71, 1.14)	.33	1.00	0.79 (0.63, 0.99)	0.90 (0.71, 1.14)	0.74 (0.57, 0.95)	.05	1.00	1.18 (0.94, 1.49)	1.12 (0.89, 1.41)	1.26 (0.99, 1.59)	.08

Abbreviations: DBP, diastolic blood pressure; FBS, fasting blood sugar; HDL, high‐density lipoprotein; hPDI, healthy plant‐based diet index; LDL, low‐density lipoprotein; PDI, plant‐based diet index; SBP, systolic blood pressure; uPDI, unhealthy plant‐based diet index. Values are odd ratio and 95% CI, Adjusted for age, sex, total energy, physical activity, and smoking. Obtained by logistic regression.

All *p* values and *p* trends are bolded.

## 4 DISCUSSION

In the present study, participants in the highest quartile of PDI had lower FBS levels. Furthermore, adherence to hPDI was associated with lower total cholesterol levels and cholesterol‐to‐HDL ratio. However, adherence to uPDI was associated with elevated levels of FBS, total cholesterol, and LDL.

The benefits of plant‐based diets for cardiovascular health have been shown in numerous studies. A meta‐analysis by Huang et al. reported that vegetarians have lower mortality from coronary heart disease (CHD) (Huang et al., 2012). Another meta‐analysis of five cohort studies demonstrated that vegetarians had a 24% lower risk of CHD mortality compared to nonvegetarians (Key et al., 1999). The above‐mentioned studies have defined vegetarian diets as consuming no or very limited amounts of meat and its products, which mimics the PDI presented in the current study.

Recently, it has been noticed that not every plant food has the same beneficial features. Accordingly, healthy (i.e., mostly whole grains, fruits, vegetables, nuts, legumes, vegetable oils, and tea/coffee) and unhealthy (i.e., sugar‐sweetened beverages, fruit juices, refined grains, potatoes, and desserts) plant food indices have been designed. In the present study, we found that PDI and hPDI were related to lower cholesterol and FBS levels, thereby tipping the balance in favor of heart health. Nevertheless, the uPDI was related to elevated cholesterol, LDL, and FBS levels. High level of cholesterol in the circulation, especially those carried via LDL, is a major cause of heart disease. It causes the accumulation of fatty deposits within the arteries, which reduces the blood flow to the heart and other critical organs, and increases the risk of stroke or heart attack (Clarke et al., 1997). LDL is also prone to oxidation which could further complicate the situation (Holvoet, 2004). On the other hand, a constant high blood sugar level, which is usually ensued from poor food choices, could also produce the same outcomes. Studies showed that high blood sugar often goes hand in hand with elevated blood pressure and cholesterol levels (Cheung & Li, 2012).

Generally, our findings were in agreement with previous studies. Bhupathiraju et al. in a cross‐sectional study showed that adherence to the PDI and hPDI was inversely associated with LDL levels. Also, adherence to the PDI, but not hPDI, was inversely associated with FBS levels (Bhupathiraju et al., 2022). Another cross‐sectional study showed that plant‐based diets were associated with more optimal blood lipid concentrations (Martin et al., 2022). Furthermore, Shin et al. showed that the highest adherence to uPDI had 22% greater odds of dyslipidemia and 48% higher odds of hypertriglyceridemia in Korean adults (Shin & Kim, 2022). A meta‐analysis of observational studies and clinical trials revealed that adherence to vegetarian diets was associated with lower serum LDL, HDL, and total cholesterol levels (Yokoyama et al., 2017).

In addition, a cohort study on South Korean adults revealed that high adherence to the hPDI was inversely associated with the risk of dyslipidemia characterized by high levels of TG, LDL, and cholesterol. Nonetheless, adherence to the uPDI resulted in a remarkable significant increased risk of lipid disorders (Lee et al., 2021). Another cohort study in South Korea showed that the uPDI was associated with a higher risk of metabolic syndrome; however, participants with the highest adherence to the PDI had a lower FBS level (Kim et al., 2020). Furthermore, a case–control study by Zamani et al. following an overall plant‐based diet was associated with a lower risk of gestational diabetes (Zamani et al., 2019).

Several mechanisms have been introduced to justify the above‐mentioned findings, most of which center around the beneficial components of plant‐based diets. Similar to the case in the present study, greater adherence to the PDI and hPDI often marks a dietary plan high in fiber, antioxidants, unsaturated fats, and some micronutrients. Dietary fiber could decrease glucose absorption and impose a beneficial effect on glucose metabolism. It also enhances cholesterol removal by binding cholesterol and bile acids (Brown et al., 1999). Other nutrients, such as vitamin C and magnesium, could also increase insulin sensitivity which resulted in better glycemic control. Moreover, high amounts of polyphenols in healthy plant foods improve the lipid profile by inhibiting the oxidation of LDL (Quiñones et al., 2012). Additionally, decreased intake of animal‐based foods, associated with higher adherence to all three plant‐based indices in this study, results in a consequent low intake of saturated fat and heme iron, which could also justify the benefits of the PDI and hPDI on FBS and lipid profile (de Oliveira Otto et al., 2012).

On the other hand, adherence to the uPDI usually results in lower consumption of fibers and antioxidants, including vitamins C and E and beta carotene, which were particularly lower in the participants with the highest adherence to the uPDI. Low consumption of antioxidants could lead to endothelial dysfunction and oxidative stress, which ultimately pave the way for the development of CVDs (Liu et al., 2005). Moreover, the regular consumption of added sugars, another common characteristic of the uPDI, can lead to poor glycemic control and lipid metabolism (Fried & Rao, 2003).

However, the present study failed to find a significant correlation between none of the plant‐based diet indices with blood pressure, TG, and HDL, among which the lack of association with blood pressure could be the most controversial factor in previous studies. Clinical trial studies abound as to the efficacy of plant‐based diets in lowering blood pressure (Crimarco et al., 2020; Jenkins et al., 2008; Rouse et al., 1986).

By recruiting a large number of participants and using validated questionnaires, the present study could further broaden the understanding of how plant‐based indices interact with single individual CVD risk factors. However, the current study suffers from some limitations. First, the recall bias in reporting dietary intake can affect the results. The cross‐sectional nature of this study was another limitation as it prevented from inferring causality. Moreover, there might have been some other potential confounders affecting the results which could not be measured or adjusted, such as lack of oxidized LDL measurement and the information on medicine intake.

## 5 CONCLUSION

The present study demonstrated that adherence to the PDI and hPDI was associated with more optimal blood glucose, cholesterol levels, and cholesterol to HDL ratio. Nevertheless, the uPDI was related to increased FBS, cholesterol, and LDL levels. The findings of this study further support the knowledge regarding the benefits of dietary patterns mainly composed of healthy plant‐based foods, such as whole grains and vegetables, while discouraging the regular intake of unhealthy plant foods, including refined grains and sugar‐sweetened beverages.

## CONFLICT OF INTEREST

All authors declare that they have no conflict of interest.

## Data Availability

Data available on request from the authors.
